# *Trichostrongylus colubriformis* Nematode Infections in Humans, France

**DOI:** 10.3201/eid1707.101519

**Published:** 2011-07

**Authors:** Stéphanie Lattès, Hubert Ferté, Pascal Delaunay, Jérôme Depaquit, Matteo Vassallo, Mélanie Vittier, Sahare Kokcha, Eric Coulibaly, Pierre Marty

**Affiliations:** Author affiliations: Centre Hospitalier Universitaire de Nice, Nice, France (S. Lattès, P. Delaunay, M. Vassallo, S. Kochka);; Université de Reims Champagne–Ardenne, Reims, France (H. Ferté, J. Depaquit, M. Vittier);; Services Vétérinaires des Alpes-Maritimes, Sophia-Antipolis, France (E. Coulibaly);; Université de Nice–Sophia Antipolis–Inserm U895, Nice (P. Marty)

**Keywords:** Trichostrongylus colubriformis, trichostrongyliasis, familial infection, human, parasites, helminth, sheep manure, France, letter

**To the Editor:** In April 2009, a 47-year-old woman in Saint-Jeannet in southern France reported stomach aches, abdominal bloating, and occasional diarrhea. Blood analyses found an increased eosinophil level (8,800 cells/mm^3^), which represented 52% of 16,900 leukocytes/mm^3^.

Parasitologic examinations for helminths were conducted with 6 fecal specimens obtained during June 9–July 2, 2009. Analyses included direct wet mount microscopic examination, Merthiolate–iodine–formaldehyde concentration, formalin–ethyl acetate concentration, and Baermann larval extraction.

Results of direct examination and the Baermann technique were negative for all samples. The formalin–ethyl acetate concentration technique detected a parasite egg (Figure, panel A) and first-stage larvae. Fecal cultures grew mature third-stage larvae (length 700–800 µm, 16 intestinal cells, length of the sheath <40 µm), belonging to the genus *Trichostrongylus* (Figure, panel B). Because of the ambiguous morphologic features of this genus, a molecular approach was necessary for specific identification (*1**,**2*).

Identical symptoms developed in 2 children of the patient and in 2 friends. The mother of the patient had additional symptoms (weight loss 5 kg in 1 month and 35,000 eosinophils/mm^3^, which represented 85% of 43,200 leukocytes/mm^3^). However, results of fecal examinations were negative for these 5 persons.

DNA was extracted separately from 2 third-stage larvae (Figure, panel B) by using the DNA Tissue Mini Kit (QIAGEN, Hilden, Germany). To amplify internal transcribed spacer 2 (ITS2) sequences, we used primers NC1: 5′-ACGTCTGGTTCAGGGTTGTT-3′ (forward) and NC2: 5′-TTAGTTTCTTTTCCTCCGCT-3′ (reverse) (*3**,**4*), which were used by Hoste et al. for *Trichostrongylus* spp. typing (*3*). ITS2 rDNA was sequenced, and third-stage larvae sequences were registered in GenBank (accession nos. HQ174256 and HQ174257).

**Figure:**
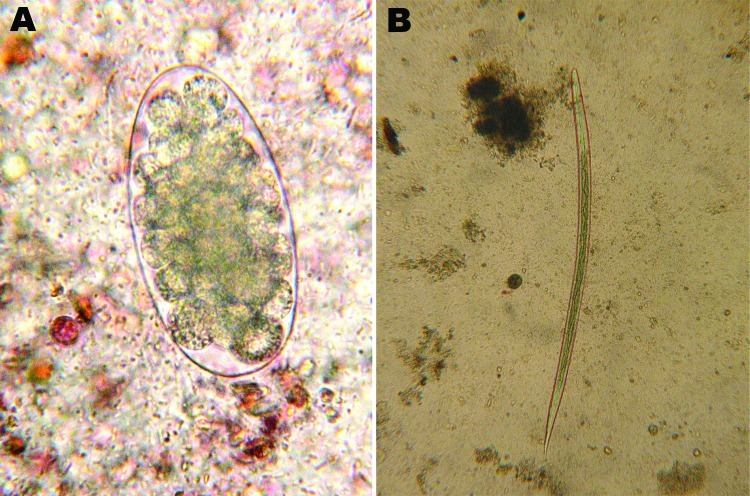
*Trichostrongylus colubriformis* nematode isolated from feces of a 47-year-old woman, France. A) Egg (length 89 µm) isolated by using direct examination (original magnification ×200). B) Third-stage larvae (length 740 µm, 16 intestinal cells, length of distal part of the sheath <40 µm) isolated by using fecal culture (original magnification ×50).

Complete (100%) homology was obtained with known sequences (*3**,**4*) for adult *Trichostrongylus colubriformis* nematodes from sheep (GenBank accession nos. S69220, X78063, and EF427624). Parasite sequences also showed 100% homology with the main haplotype observed in humans in Laos (*2*). If one considers the absence of intraspecific variability within *T. colubriformis* nematodes (*3**,**4*), the specimens isolated from the patient and most likely from the other 5 persons presumed to be affected in this outbreak belong to this species.

The 6 symptomatic patients were treated according to published recommendations (*5*) with albendazole, 400 mg/day for 10 days. Clinical remission was obtained in <3 days, and eosinophil counts returned to reference levels 3 months later.

Specific questioning of the 6 persons indicated that the source of infection most likely was a meal eaten in April 2009, which included strawberries picked in the vegetable garden of the patient’s mother. The patient’s father and brother did not eat any strawberries and did not have any symptoms. The garden was fertilized yearly with dried manure from a local sheep farm. Lack of dried manure in 2009 led to use of fresh sheep manure from the same farm. Sheep manure from breeding stock on the farm was examined. *Trichostrongylus* spp. third-stage larvae were found despite prophylactic treatment of sheep on the farm against helminths.

*T. colubriformis* nematodes are mainly parasites of herbivorous mammals and have a worldwide distribution. Human infections are found predominantly in warm areas. They are usually asymptomatic or as described in the present case. *T. colubriformis* adults live in the intestines of the host (*6*). The female lays eggs, which are excreted in feces. Eggs then hatch and mature into infectious larvae. Humans become infected by ingesting unwashed vegetables contaminated by animal feces containing strongyloid larvae. Larvae mature into adults in the intestines.

Sporadic cases of this infection in humans have been reported in many countries (*7*). In France, several autochthonous cases were suspected, but because of their rarity and difficulty in identification, they are not commonly reported (*8*). Eggs of *Trichostrongylus* spp. can be differentiated from those of *Necator* and *Ancylostoma* spp. because they are longer, narrower, and elongated. After 6 days of culture, *T*. *colubriformis* nematodes can be distinguished from similar stages in *Strongyloides* and *Ancylostoma* spp. by the bead-like swelling at the tip of the tail. Except for isolation of adult worms, which are rarely found in feces, sequencing of the ITS2 region is the most accurate method for specific identification of *Trichostrongylus* spp. isolated from humans.

This familial outbreak highlights increased risk for animal parasitosis in humans in an industrialized country, which may have been caused by an increasing trend of persons using ecologic and organic farming methods. These cases confirm that hygienic recommendations for use of organic fertilizer must be disseminated on a large scale. It is also mandatory that fresh vegetables be washed carefully and thoroughly before ingestion, and only dried manure should be used as an organic fertilizer.
